# HPV genotyping of cervical histologic specimens of 61, 422 patients from the largest women hospital in China

**DOI:** 10.3389/fonc.2023.1161631

**Published:** 2023-03-29

**Authors:** Fangfang Zhong, Zaibo Li, Yihua Sun, Yaoxing Xiao, Juan Li, Xianrong Zhou, Qing Cong, Long Sui, Xiang Tao, Chengquan Zhao

**Affiliations:** ^1^ Department of Pathology, Obstetrics and Gynecology Hospital of Fudan University, Shanghai, China; ^2^ Department of Pathology, The Ohio State University, Columbus, OH, United States; ^3^ Department of Pathology, Jinan Maternity and Child Care Hospital, Jinan, Shandong, China; ^4^ Center for Diagnosis and Treatment of Cervical Diseases, Obstetrics and Gynecology Hospital of Fudan University, Shanghai, China; ^5^ Department of Pathology, University of Pittsburgh Medical Center, Pittsburgh, PA, United States

**Keywords:** human papillomavirus, genotyping, cervical neoplasia, cervical cancer, China

## Abstract

**Objectives:**

We investigated HPV genotypes in a large cohort of patients with definitive cervical histologic diagnosis.

**Methods:**

HPV testing was performed by real-time PCR assay, including 18 high-risk HPV (hrHPV) and 3 low-risk HPV (lrHPV). Totally 61,422 patients with documented HPV genotyping results within 6 months before cervical histologic diagnoses were included.

**Results:**

HrHPV positive rate was 55.1% among all tested cases with the highest in adenosquamous carcinoma (94.1%), followed by squamous cell carcinoma (SCC) (93.7%), cervical intraepithelial neoplasia 2/3 (CIN2/3) (92.8%). HrHPV positive rates were significantly higher in high-grade squamous lesions than in those in glandular lesions. HPV16 was the most common genotype followed by HPV52 and HPV58 in CIN2/3. The most frequent hrHPV genotype in adenocarcinoma *in situ* (AIS) was HPV18, followed by HPV16, HPV45 and HPV52. In SCC cases, HPV16 was the most common type followed by HPV58, HPV52, HPV18 and HPV33. However, HPV18 showed significantly higher prevalence in adenocarcinoma and adenosquamous carcinoma than in that in SCC. Theoretically, the protective rates of 2/4-valent and 9-valent vaccine were 69.1% and 85.8% for cervical cancers.

**Conclusions:**

The prevalence of HPV genotypes in Chinese population was different from that in Western population. Some hrHPV types were identified in cervical precancerous lesions and cancers, which are not included in current HPV vaccines. These data provide baseline knowledge for future HPV vaccine development.

## Introduction

Cervical cancer is the second most common type of malignancies among women and was responsible for 341,000 deaths in 2020 with approximately 80% in the developing countries (1). Most cervical cancers are caused by human papillomavirus (HPV) infection. HPV is a DNA virus comprising more than 100 genotypes. Thirteen high-risk HPV (hrHPV) types including type 16, 18, 31, 33, 35, 39, 45, 51, 52, 56, 58, 59, and 68, account for more than 90% of cervical cancers, with HPV16 and HPV18 being the most common oncogenic genotypes. Clinically, hrHPV status is usually determined by HPV pooled assays with limited genotyping (HPV16 and HPV18) (2). Due to variable prevalence of hrHPV in different populations, hrHPV assays with full spectrum of genotypes is superior to assays with limited genotyping (3).

The prevalence of different genotypes of hrHPV has been mostly studied in Western populations (4, 5), but not in Chinese population. HPV genotyping can provide the baseline hrHPV infection, thus supply a reference to evaluate the protective effects of vaccination with different genotypes of hrHPV. Currently available HPV genotyping studies were mostly regional and relatively small in size (6–8), and their cohorts were stratified according to cytology interpretations which included some non-definitive diagnosis such as atypical squamous cells (ASC) of undetermined significance (ASC-US), ASC cannot exclude an high-grade squamous intraepithelial lesions (ASC-H) and atypical glandular cells (AGC) etc. In this study, we presented a large cohort of 61,422 patients with definitive cervical histologic diagnosis and HPV genotyping results from HPV assays with broad spectrum of genotypes from the largest academic women’s hospital in China, covering a large population in East China.

## Materials and methods

### Patient cohort

The study cohort included 61,422 women with cervical histologic diagnoses and recent HPV results with extended genotyping (≤ 6 months before histologic diagnosis) between October 2017 and May 2022 from Obstetrics and Gynecology Hospital of Fudan University (OGHFU). OGHFU is the largest women’s hospital and academic center in China and serves a variety of patient populations from Shanghai city, adjacent counties and cities, and nationwide referrals. Histologic specimens included colposcopic punch biopsies, endocervical curettages, cone biopsies, and hysterectomies. The worst diagnosis was recorded if the patient had multiple cervical histologic diagnoses. Cases with endometrial carcinoma extending to uterine cervix, metastatic carcinoma, sarcoma and melanoma were excluded. Only cases with documented prior HPV genotyping results within 6 months before cervical histologic diagnoses were included in this study. This study was approved by the Institutional Review Boards of OGHFU.

### HPV genotyping

HPV extended genotyping was performed by utilizing the BioPerfectus Multiplex Real-Time PCR assay (BMRT) assay (BioPerfectus Technology Co, Taizhou, China), detecting 21 HPV genotypes, including 18 high-risk HPV (hrHPV) and 3 low-risk HPV (lrHPV) (9). The detection method has been validated and approved by the China Food and Drug Administration (CFDA). This assay was carried out according to the manufacturer’s protocol using cervical liquid cytology samples which were collected by gynecologists and stored in standard preservative media provided by manufacturers. Briefly, PCR primers and corresponding TaqMan probes were used to detect each of the 21 most prevalent HPV types, including 18 HR and possible HR HPV genotypes (HPV-16, -18, -26, -31, -33, -35, -39, -45, -51, -52, -53, -56, -58, -59, -66, -68, -73 and -82) and 3 low-risk HPV (LR-HPV) genotypes (HPV-6, -11 and -81 (equivalent to CP8304)). Results were rendered as negative or positive, with viral loads per cell for each positive genotype.

### Histologic diagnosis

Subspecialized gynecologic pathologists at OGHFU signed out all histologic pathology reports according to the World Health Organization (WHO) classification (10). The diagnosis includes negative for intraepithelial lesions or malignancies (negative), cervical intraepithelial neoplasia 1 (CIN1), CIN2/3, endocervical adenocarcinoma *in situ* (AIS), AIS with CIN2/3 (AIS+CIN2/3), squamous cell carcinoma (SCC), endocervical adenocarcinoma (ADC) including usual type, gastric type, etc., adenosquamous carcinoma (ADSQ) and small cell neuroendocrine carcinoma (SNEC).

### Statistical analysis

Student’s *t* test was performed for 2 groups and analysis of variance (ANOVA) was performed for more than 2 groups. The differences between ratios were analyzed by Pearson *χ *(2) test or Fisher Exact test. A p value of ≤0.05 was considered the criterion for statistical significance.

## Results

### Study population

The study cohort consisted of 61,422 cases, including 46,440 negative (75.6%), 10,058 CIN1 (16.4%), 3,773 CIN2/3 (6.1%), 778 SCC (1.3%), 57 AIS (0.1%), 111 ADC (0.18%), 87 AIS+CIN2/3 (0.14%), 101 ADSQ and 17 SNEC (0.03%) cases (**Table 1**). The mean age of women was 42.5 years old (range, 15-92 years). The mean age increased as the squamous lesions advanced (ANOVA test, *P*<0.001). The mean ages of ADC and ADSQ were younger than that of SCC (ANOVA test, *P*<0.001) (**Table 1**).

**Table 1:** Prevalence of HPV infection within various cervical histological diagnoses.

Histology	Case#	Mean Age (ranges)	hrHPV		lrHPV+	
			Case#	%	Case#	%
**Negative**	46,440	43.0 (15-92)	20,396	43.9	2682	5.8
**CIN1**	10,058	39.1 (15-81)	8,896	88.4	1465	14.6
**CIN2/3**	3,773	42.7 (16-85)	3,502	92.8	239	6.3
**SCC**	778	52.0 (22-84)	729	93.7	32	4.1
**AIS**	57	39.6 (25-79)	50	87.7	2	3.5
**ADC**	111	47.9 (18-82)	78	70.3	1	0.9
**AIS+ CIN2/3**	87	38.9 (21-70)	80	92.0	5	5.7
**ADSQ**	101	48.6 (23-71)	95	94.1	4	4.0
**SNEC**	17	43.4 (22-64)	14	82.4	1	5.9
**Total**	61,422	42.5 (15-92)	33,840	55.1	4431	7.2

CIN1, cervical intraepithelial neoplasia 1; CIN2/3, cervical intraepithelial neoplasia 2/3; AIS, endocervical adenocarcinoma *in situ*; SCC, squamous cell carcinoma of cervix, ADC, invasive adenocarcinoma of endocervix; ADSQ, adenosquamous carcinoma of endocervix; SNEC, small cell neuroendocrine carcinoma of endocervix; hrHPV, high risk human papillomavirus; lrHPV, low risk human papillomavirus; #, number.

18 genotypes of hrHPV, as detected by the BioPerfectus Multiplex Real-Time PCR assay in current study, include HPV-16, HPV-18, HPV-31, HPV-33, HPV-35, HPV-39, HPV-45, HPV-51, HPV-52, HPV-56, HPV-58, HPV-59, HPV-66, HPV-68, HPV-26, HPV-53, HPV-73, and HPV-82. Three genotypes of lrHPV, as detected by the BioPerfectus Multiplex Real-Time PCR assay, including HPV-6, HPV-11, and HPV-81.

### HPV prevalence

The median of the time between HPV testing and histopathologic diagnoses was 24 days (quartiles 10, 41). 62.7% of cases had an HPV test within one month prior to cervical histopathologic diagnoses. Most patients with extended HPV genotyping also had a companying cervical cytology test at OGHFU. The current study was aimed to investigate hrHPV genotyping results in various cervical histological findings; therefore, cervical cytology test results were not included.

HrHPV positive rate was 55.1% among all tested cases with the highest in ADSQ (94.1%), followed by SCC (93.7%), CIN2/3 (92.8%), CIN2/3+AIS (92%) and CIN1 (88.4%). HrHPV positive rates were significantly higher in high grade squamous lesions than those in glandular lesions (**Table 1**). When only 14 hrHPV genotypes (HPV-16, HPV-18, HPV-31, HPV-33, HPV-35, HPV-39, HPV-45, HPV-51, HPV-52, HPV-56, HPV-58, HPV-59, HPV-66, and HPV-68) as included in other commercial HPV assays such as Cobas4800 or Aptima were counted, hrHPV positive rate was slightly decreased to 51.8%. Overall lrHPV detection rate was 7.2% with the highest positive rate in CIN1 cases (14.6%) (**Table 1**). Among 4431 lrHPV positive cases, 3125 cases (70.5%) had hrHPV coinfection. Only lrHPV infection case was found in 1 of 111 ADC cases, but not in other types of cancers or AIS.

### HrHPV prevalence in patients with different age groups

All cases were stratified into 3 age groups: < 30 years, 30 to 49 years, and 50 years and above. HrHPV prevalence was highest in the <30 group in all categories except SCC and ADC. HrHPV prevalence was remarkably lower in 50 years and older group with glandular lesions (ADC and AIS) than in 30-49 years group (*P*<0.001) (**Table 2**).

**Table 2:** Prevalence of hrHPV infection within various histological groups by age.

Histology	<30 y		30-49 y		≥50 y	
	Cases#	Positive (%)	Cases#	Positive (%)	Cases#	Positive (%)
**Negative**	5,883	3,422 (58.2)	27,008	11040 (40.9)	13,549	5934 (43.8)
**CIN1**	2,396	2,174 (90.7)	5,587	4909 (87.9)	2,075	1813 (87.4)
**CIN2/3**	510	481 (94.3)	2198	2040 (92.8)	1,065	981 (92.1)
**SCC**	14	12 (85.7)	292	269 (92.1)	472	448 (94.9)
**AIS**	5	5 (100.0)	44	39 (88.6)	8	6 (75.0)
**ADC**	6	3 (50.0)	57	47 (82.5)	48	28 (58.3)
**AIS+ CIN2/3**	18	18 (100.0)	52	48 (92.3)	17	14 (82.4)
**ADSQ**	6	6 (100.0)	44	41 (93.2)	51	48 (94.1)
**SNEC**	2	2 (100.0)	9	9 (100.0)	6	3 (50.0)

CIN1, cervical intraepithelial neoplasia 1; CIN2/3, cervical intraepithelial neoplasia 2/3; AIS, endocervical adenocarcinoma *in situ*; SCC, squamous cell carcinoma of cervix, ADC, invasive adenocarcinoma of endocervix; ADSQ, adenosquamous carcinoma of endocervix; SNEC, small cell neuroendocrine carcinoma of endocervix; hrHPV, high-risk human papillomavirus; #, number; y, years.

### Simultaneous multiple hrHPV co-infection

We further analyzed multiple hrHPV infection (≥2 types) in different age and histologic groups. Overall, multiple hrHPV infection occurred in 30.5% of hrHPV-positive cases (10,322/33,840), with up to 10 different hrHPV genotypes in a single case. CIN1 cases showed the highest percentage of multiple infection at 41.3% (3672/8896), while ADSQ cases had the lowest at 16.8% (16/95). Multiple infection occurred more frequently in young patients (<30 years old) than in older patients, and also more frequently in squamous lesions (CINs and cancers) than in glandular lesions (AIS and carcinomas) (38.4% vs. 21.8%) (**Table 3**).

**Table 3:** Percentage of multiple hrHPV infection among hrHPV-positive cases within various histological groups.

Histology	<30 y		30-49 y		≥50 y		Total	
	hrHPV Positive#	2≥hrHPV# (%)	hrHPV Positive#	≥2 hrHPV# (%)	hrHPV Positive#	≥2 hrHPV# (%)	hrHPV Positive#	≥2 hrHPV# (%)
**Negative**	3,422	1040 (30.4)	11,040	2,412 (21.8)	5,934	1760 (29.7)	20,396	5212 (25.6)
**CIN1**	2,174	1063 (48.9)	4,909	1,764 (35.9)	1813	845 (46.6)	8,896	3672 (41.3)
**CIN2/3**	481	191 (39.7)	2,040	634 (31.1)	981	376 (38.3)	3,502	1201 (34.3)
**SCC**	12	2 (16.7)	269	52 (19.3)	448	116 (25.9)	729	170 (23.3)
**AIS**	5	0 (0.0)	39	5 (12.8)	6	0 (0.0)	50	5 (10.0)
**ADC**	3	2 (66.7)	47	12 (25.5)	28	7 (25.0)	78	21 (26.9)
**AIS+HSIL**	18	5 (27.8)	48	12 (25.0)	14	3 (21.4)	80	20 (25.0)
**ADSQ**	6	1 (16.7)	41	3 (7.3)	48	12 (25.0)	95	16 (16.8)
**SNEC**	2	1 (50.0)	9	4 (44.4)	3	0 (0.0)	14	5 (35.7)

CIN1, cervical intraepithelial neoplasia 1; CIN2/3, cervical intraepithelial neoplasia 2/3; AIS, endocervical adenocarcinoma *in situ*; SCC, squamous cell carcinoma of cervix, ADC, invasive adenocarcinoma of endocervix; ADSQ, adenosquamous carcinoma of endocervix; SNEC, small cell neuroendocrine carcinoma of endocervix; hrHPV, high-risk human papillomavirus; #, number; y, years.

### Distribution of hrHPV genotypes in patients with precancerous lesion or negative histologic result

**Figure 1:**
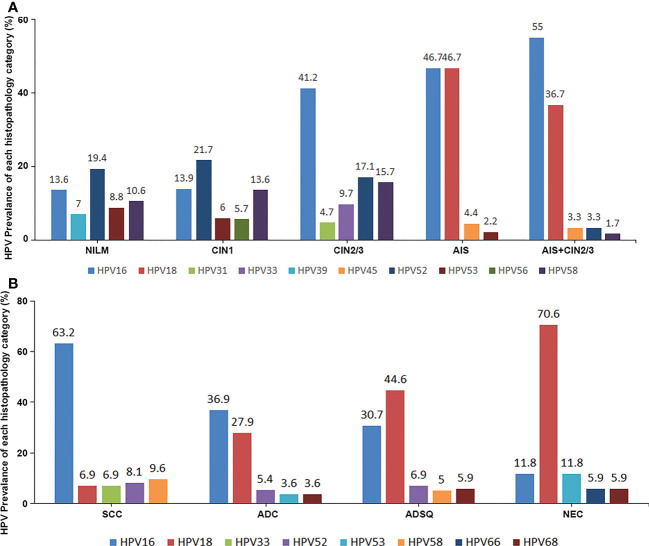
The top 5 most prevalent hrHPV genotypes in each lesional group, as well as the negative control, in both the precancerous group **(A)** and the cancer group **(B)**.

The distribution of all 18 genotypes of hrHPV in patients with precancerous lesion or negative histologic result were summarized. HrHPV genotypes were more evenly distributed in negative cases than in precancerous lesions, with HPV52 (10.1%) being the most common genotype, followed by HPV16 (7.1%), HPV53 (5.9%), HPV58 (5.9%), and HPV39 (4.2%). CIN1 cases and negative cases shared similar patterns of HPV genotyping results but showed higher hrHPV positive rates with HPV52 (25.2%) as the most common genotype, followed by HPV16 (16.9%), HPV58 (16.5%), HPV53 (12.1%), and HPV56 (9.5%). In CIN2/3 precancerous lesions, HPV16 was the most common genotype (40.1%) followed by HPV31 (7.3%) and HPV33 (12.1%). The distribution of hrHPV genotypes was more limited in AIS than in squamous precancerous lesions and negative cases. The most frequent hrHPV genotype in AIS was HPV18 (42.1%) followed by HPV16 (40.4%), HPV45 (5.3%), HPV52 (5.3%), and HPV53, 58, 59 (each with 1.8%). The other 10 hrHPV types were not detected in the AIS group. The pattern of hrHPV genotyping of AIS/CIN2/3 was similar to AIS and showed significantly more HPV18 and HPV45 infections than CIN2/3. In comparison with negative/CIN1 cases, CIN2+ (CIN2/3, AIS, AIS/CIN2/3) cases demonstrated significantly higher positive rates for HPV16 (40.3% vs 8.8%), HPV31 (7.1% vs 2.9%), HPV33 (11.75 vs 2.7%), HPV52 (22.2% vs 12.8%) and HPV58 (18.8% vs 7.7%) (**Table 4**; **Figure 1A**).

**Table 4:** hrHPV genotypes for cases with negative and various types of cervical intraepithelial neoplasia histologically.

HPV Types	≤ CIN1			CIN2+			
	Negative (n=46440) Positive (%)	CIN1 (n=10058) Positive (%)	Total (n=56498) Positive (%)	CIN2/3 (n=3773) Positive (%)	AIS (n=57) Positive (%)	AIS/CIN2/3 (n=87) Positive (%)	Total (n=3917) Positive (%)
HPV16	3290 (7.1)	1698 (16.9)	**4988 (8.8)**	1513 (40.1)	23 (40.4)	44 (50.6)	**1580 (40.3)**
HPV18	1131 (2.4)	692 (6.9)	**1823 (3.2)**	140 (3.7)	24 (42.1)	29 (33.3)	**193 (4.9)**
HPV26	84 (0.2)	59 (0.6)	**143 (0.3)**	24 (0.6)	0 (0.0)	0 (0.0)	**24 (0.6)**
HPV31	1047 (2.3)	597 (5.9)	**1644 (2.9)**	277 (7.3)	0 (0.0)	0 (0.0)	**277 (7.1)**
HPV33	943 (2.0)	582 (5.8)	**1525 (2.7)**	457 (12.1)	0 (0.0)	0 (0.0)	**457 (11.7)**
HPV35	600 (1.3)	335 (3.3)	**935 (1.7)**	109 (2.9)	0 (0.0)	1 (1.1)	**110 (2.8)**
HPV39	1961 (4.2)	888 (8.8)	**2849 (5.0)**	146 (3.9)	0 (0.0)	0 (0.0)	**146 (3.7)**
HPV45	337 (0.7)	186 (1.8)	**523 (0.9)**	44 (1.2)	3 (5.3)	4 (4.6)	**51 (1.3)**
HPV51	1491 (3.2)	892 (8.9)	**2383 (4.2)**	158 (4.2)	0 (0.0)	4 (4.6)	**162 (4.1)**
HPV52	4710 (10.1)	2533 (25.2)	**7243 (12.8)**	858 (22.7)	3 (5.3)	8 (9.2)	**869 (22.2)**
HPV53	2729 (5.9)	1216 (12.1)	**3945 (7.0)**	207 (5.5)	1 (1.8)	2 (2.3)	**210 (5.4)**
HPV56	1759 (3.8)	960 (9.5)	**2719 (4.8)**	152 (4.0)	0 (0.0)	1 (1.1)	**153 (3.9)**
HPV58	2718 (5.9)	1658 (16.5)	**4376 (7.7)**	730 (19.3)	1 (1.8)	4 (4.6)	**735 (18.8)**
HPV59	1317 (2.8)	680 (6.8)	**1997 (3.5)**	109 (2.9)	1 (1.8)	2 (2.3)	**112 (2.9)**
HPV66	1482 (3.2)	784 (7.8)	**2266 (4.0)**	138 (3.7)	0 (0.0)	5 (5.7)	**143 (3.7)**
HPV68	1589 (3.4)	696 (6.9)	**2285 (4.0)**	147 (3.9)	1 (1.8)	1 (1.1)	**149 (3.8)**
HPV73	228 (0.5)	132 (1.3)	**360 (0.6)**	34 (0.9)	0 (0.0)	0 (0.0)	**34 (0.9)**
HPV82	235 (0.5)	156 (1.6)	**391 (0.7)**	77 (2.0)	0 (0.0)	1 (1.1)	**78 (2.0)**

CIN1, cervical intraepithelial neoplasia 1; CIN2/3, cervical intraepithelial neoplasia 2/3; AIS, endocervical adenocarcinoma *in situ*; SCC, squamous cell carcinoma of cervix, ADC, invasive adenocarcinoma of endocervix; ADSQ, adenosquamous carcinoma of endocervix; SNEC, small cell neuroendocrine carcinoma of endocervix; hrHPV, high-risk human papillomavirus; n, number.

The bold font represented the total data of each item.

### Distribution of hrHPV genotypes in patients with cervical carcinomas

The distribution of hrHPV genotypes in cervical cancer cases was more concentrated than that in precancerous cases. The genotyping of 1,007 cases of cervical cancers showed that HPV16 accounted for 56.2% of cases, HPV18 for 14.1%, HPV58 for 8.0%, HPV52 for 7.5%, and HPV33 for 5.9%. For SCC cases, the prevalence of HPV16 was 63.2%, followed by HPV58 (9.6%), HPV52 (8.1%), and HPV33 (6.9%). HPV18 showed significantly higher prevalence in ADC (27.9%), ADSQ (44.6%), and SNEC (70.6%) than in SCC. Four potential hrHPV genotypes, HPV53, HPV26, HPV82, and HPV73, were not included in FDA-approved commercial kits, which showed positive rates of 4.2%, 1.0%, 0.9%, and 0.2%, respectively (**Table 5**; **Figure 1B**).

**Table 5:** hrHPV genotypes for 1007 cases of cervical cancer.

HPV Types	SCC (n=778) Positive (%)	ADC (n=111) Positive (%)	ADSQ (n=101) Positive (%)	SNEC (n=17) Positive (%)	Total (n=1007) Positive (%)
HPV16	492 (63.2)	41 (36.9)	31 (30.7)	2 (11.8)	**566 (56.2)**
HPV18	54 (6.9)	31 (27.9)	45 (44.6)	12 (70.6)	**142 (14.1)**
HPV26	9 (1.2)	1 (0.9)	0 (0.0)	0 (0.0)	**10 (1.0)**
HPV31	39 (5.0)	1 (0.9)	2 (2.0)	0 (0.0)	**42 (4.2)**
HPV33	54 (6.9)	2 (1.8)	3 (3.0)	0 (0.0)	**59 (5.9)**
HPV35	10 (1.3)	1 (0.9)	1 (1.0)	0 (0.0)	**12 (1.2)**
HPV39	18 (2.3)	2 (1.8)	1 (1.0)	1 (5.9)	**22 (2.2)**
HPV45	11 (1.4)	2 (1.8)	4 (4.0)	0 (0.0)	**17 (1.7)**
HPV51	12 (1.5)	0 (0.0)	1 (1.0)	0 (0.0)	**13 (1.3)**
HPV52	63 (8.1)	6 (5.4)	7 (6.9)	0 (0.0)	**76 (7.5)**
HPV53	35 (4.5)	4 (3.6)	1 (1.0)	2 (11.8)	**42 (4.2)**
HPV56	30 (3.9)	1 (0.9)	4 (4.0)	1 (5.9)	**36 (3.6)**
HPV58	75 (9.6)	1 (0.9)	5 (5.0)	0 (0.0)	**81 (8.0)**
HPV59	23 (3.0)	3 (2.7)	4 (4.0)	0 (0.0)	**30 (3.0)**
HPV66	17 (2.2)	2 (1.8)	0 (0.0)	1 (5.9)	**20 (2.0)**
HPV68	20 (2.6)	4 (3.6)	6 (5.9)	1 (5.9)	**31 (3.1)**
HPV73	2 (0.3)	0 (0.0)	0 (0.0)	0 (0.0)	**2 (0.2)**
HPV82	5 (0.6)	0 (0.0)	4 (4.0)	0 (0.0)	**9 (0.9)**

CIN1, cervical intraepithelial neoplasia 1; CIN2/3, cervical intraepithelial neoplasia 2/3; AIS, endocervical adenocarcinoma *in situ*; SCC, squamous cell carcinoma of cervix, ADC, invasive adenocarcinoma of endocervix; ADSQ, adenosquamous carcinoma of endocervix; SNEC, small cell neuroendocrine carcinoma of endocervix; hrHPV, high-risk human papillomavirus; n, number.

The bold font represented the total data of each item.

### The protection of the Bivalent/quadrivalent and the 9-valent HPV vaccines

Chinese population were generally unvaccinated by hrHPV vaccine, so we explored the baseline HPV genotypes of various precancers and cancers, to evaluate the protective effect of 2/4-valent (HPV16/18 plus HPV6/11) and 9-valent (HPV16/18/31/33/45/52/58 plus HPV6/11) commercially available vaccine. For precancerous lesions, the possible coverage of 2/4-valent and 9-valent vaccines were 43.1% (1,626/3773), 86.0% (3,245/3,773) for CIN2/3, respectively. For AIS, the coverage of 2/4-valent was higher than those of CIN2/3, reaching to 82.5% (47/57). While the coverage of 9-valent vaccines was the same with 2/4-valent vaccines, both of which were 86.0%. The rates of AIS accompanied by CIN2/3 were higher than the above two lesions. In malignancies, the coverage of 2/4-valent for SCC, ADC, ADSQ, and SNEC were 68.8%, 64.0%, 75.2%, and 84.2%, respectively. The percentages for 9-valent were 87.9%, 67.6%, 90.1%, and 82.4%, respectively. The total protective rate for 9-valent vaccine were 49.6% (2,441/4,924) and 86.1% (4,237/4,924) for precancerous lesions and cancer theoretically (**Table 6**).

**Table 6:** The protection of the Bivalent/quadrivalent and the 9-valent HPV vaccines for cervical precancer lesions and cancers in Chinese population.

Cervical lesions	hrHPV positive case	HPV16/18		HPV16/18/31/33/45/52/58	
		Positive case	Percentage	Positive case	Percentage
CIN2/3	3,773	1626	43.1	3245	86.0
AIS	57	47	82.5	49	86.0
AIS+CIN2/3	87	72	82.8	79	90.8
**Summary**	**3,917**	**1,745**	**44.5**	**3,373**	**86.1**
SCC	778	535	68.8	684	87.9
ADC	111	71	64	75	67.6
ADSQ	101	76	75.2	91	90.1
SNEC	17	14	82.4	14	82.4
**Summary**	**1,007**	**696**	**69.1**	**864**	**85.8**

CIN2/3, cervical intraepithelial neoplasia 2/3; AIS, endocervical adenocarcinoma *in situ*; SCC, squamous cell carcinoma of cervix, ADC, invasive adenocarcinoma of endocervix; ADSQ, adenosquamous carcinoma of endocervix; SNEC, small cell neuroendocrine carcinoma of endocervix; hrHPV, high-risk human papillomavirus.

The bold font represented the total data of each item.

## Discussion

Although more than 100 genotypes of HPV can infect humans, only 25 HPV types (16, 18, 26, 30, 31, 33, 34, 35, 39, 45, 51, 52, 53, 56, 58, 59, 66, 67, 68, 69, 70, 73, 82, 85, and 97) are considered high risk (oncogenic) types by the International Agency for Research on Cancer (IARC) (11). Fourteen HPV types, including 16, 18, 31, 33, 35, 39, 45, 51, 52, 56, 58, 59, 66, and 68, were the most commonly tested types in commercially available assays (12, 13). In current study, we used an extended genotyping kit including the above-mentioned 14 types and 4 additional types (26, 53, 73, and 81) to investigate the prevalence of each HPV type in different cervical histologic categories. Addition of these 4 hrHPV types resulted in an increase of hrHPV positive rate from 51.8% to 55.1%, and they are more prevalent in cervical carcinomas than some of those 14 genotypes, indicating their roles in cervical carcinogenesis and clinical utility.

The prevalence of different HPV genotypes varies among populations (14–24). Our results indicated that HPV52 and HPV58 were more prevalent in Chinese patients with negative or CIN1 histologic findings than patients from Western countries, while HPV16 and HPV33 were more frequently identified in Mexico and Norwegian populations (25, 26). HPV16 was considered as the most common HPV genotype in CIN2/3+ lesions in various populations. However, one study reported that HPV42 and HPV70 were also among the most prevalent genotypes, but they are not included in most commercially available assays (27). The population-based HPV genotype prevalence is important for vaccine development and implementation. One study from China demonstrated that 7.23% of CIN2/3 and 6.34% of SCC patients had HPV infection with genotypes which are included in the 9-valent HPV vaccine (28). Our results actually showed that up to 28.3% of CIN2/3 and 20.3% of SCC had HPV infection with genotypes other than those included in the 9-valent HPV vaccine, such as HPV51 and HPV59, which were more frequently detected than HPV45 in Chinese women. There is currently only one licensed HPV vaccine for use in the United States called Gardasil 9. Although several HPV vaccines are available in China and have been approved by the Chinese Food and Drug Administration (CFDA), including foreign HPV vaccines (Cervarix, Gardasil^®^, and Gardasil‐9^®^) and two domestic bivalent HPV vaccines, HPV vaccination rate is very low in China. In our study, the total protective rate for 9-valent vaccine were 86.1% for precancerous lesions and 85.8% for cancer theoretically, higher than that for 2- or 4-valent vaccine. However, 2 and 4-valent HPV vaccines still can prevent 70% cervical cancers. 2 or 4-valent HPV vaccination can provide benefit for primary cervical cancer prevention for the women in low source countries. Our results provided a baseline of HPV genotype in cervical precancerous lesions and cervical cancers for future HPV vaccine development for Chinese population. Additionally, HPV vaccination may cause HPV genotype prevalence change among populations with increase of HPV genotypes, which are not included in HPV vaccine (3). Four potential hrHPV genotypes, HPV53, HPV26, HPV82, and HPV73, were not included in FDA-approved commercial kits, which showed positive rates of 4.2%, 1.0%, 0.9%, and 0.2%, respectively in current study. Previous studies demonstrated that HPV82, 53 had relatively higher risk for CIN2+ lesions in women with slight abnormality

Cervical squamous and glandular lesions have distinct HPV genotyping patterns (14–17, 29). In high grade precancerous lesions, HPV16 was the most common type in CIN2/3 (40.1%), while HPV18 was the most common type in AIS (42.1%). The 2^nd^ and 3^rd^ most common types in CIN2/3 were HPV52 (22.7%) and HPV58 (19.3%), while AIS showed HPV16 (40.4%) and HPV52 (9.2%) as the 2^nd^ and 3^rd^ most common types. HPV18 was only positive in 3.7% of CIN2/3. In cervical carcinomas, the first two most common types were HPV16 (56.2%) and HPV18 (14.2%), consistent with previous studies (4, 5). In squamous cell carcinomas, the first 4 most common types were HPV16 (63.2%), HPV58 (9.6%), HPV52 (8.1%) and HPV18 (6.9%), and were HPV16 (36.9%), HPV18 (27.9%), HPV52 (5.4%) and HPV53 (3.6%) in adenocarcinomas. These findings demonstrated squamous cell carcinomas had more HPV16 and HPV58, but less HPV18 infections. One study by de Sanjosé et al. reported HPV45 as the 3^rd^ most common type in cervical adenocarcinomas after HPV18 and HPV16, accounting for up to 12% of adenocarcinomas (4). HPV45 accounted for only 1.8% of adenocarcinomas in our study cohort, consistent with another study from Western China (30), indicating different population-specific prevalence of HPV genotypes.

HPV infection with multiple genotypes is an intriguing phenomenon in cervical lesions, and its prevalence is associated with population, socioeconomic status and other factors including HPV test assay used (31–39). Spinillo et al. found that HPV multiple infection was correlated with cervical area involved by lesions, ranging from 35.7% in lesions involving less than 25% of the cervix to 59.6% in lesions involving more than 75% of the cervix (31). Kim et al. reported that HPV multiple infection was associated with persistent infection (35). Consistent with previous findings (40), HPV multiple infection was less common in SCC than precancerous lesions (23.3% in SCC vs 34/3% in CIN2/3) in our study, suggesting that clonal expansion of carcinoma cells infected by certain HPV genotype exceeds the accompanying precancerous lesions infected by other genotypes. This is supported by earlier findings that a single HPV type was preferentially expressed in invasive cervical carcinomas infected by multiple HPV genotypes (41).

Neuroendocrine carcinoma is an aggressive tumor of the cervix. In this study, 17 cervical neuroendocrine carcinomas included were found to have HPV18 infection (70.6%), which is similar to the findings of other studies with a prevalence of 75.3% to 90% (42, 43), implying that HPV18 may play a role in the tumorigenesis of such cancer.

The limitation of the current study is about the bias of the study sample. Although the sample size was large, it came from single institute, which might be different from other institutes or hospitals In addition, the patients were chosen from the hospital base, so the selection bias was present. hrHPV positive rate for ASC-US women in general population will be much lower than that in this study. Four potential hrHPV genotypes, HPV53, HPV26, HPV82, and HPV73, were not included in FDA-approved commercial kits, which showed positive rates of 4.2%, 1.0%, 0.9%, and 0.2%, respectively in current study. Previous studies demonstrated that HPV82, 53 had relatively higher risk for CIN2+ lesions in women with slight abnormality (9, 44, 45). The varieties between populations should be considered for HPV screen and HPV vaccination.

In summary, this is by far the largest study of HPV genotyping in histologically diagnosed cervical lesions from 61,422 Chinese women. Our results have demonstrated the prevalence of individual HPV genotypes in the cervical lesions in Chinese population was different from that in Western population. Furthermore, we have identified some hrHPV types in cervical precancerous lesions and cancers, which are not covered by current HPV vaccines. These data would provide baseline knowledge for future HPV vaccine development in the Chinese population.

## Data availability statement

The raw data supporting the conclusions of this article will be made available by the authors, without undue reservation.

## Ethics statement

The studies involving human participants were reviewed and approved by Institutional Review Boards of Obstetrics and Gynecology Hospital of Fudan University. Written informed consent for participation was not required for this study in accordance with the national legislation and the institutional requirements.

## Author contributions

FZ, Conceptualization, investigation, data analysis, writing the original draft; ZL, Conceptualization, data analysis, writing the original draft; YS, data analysis, review and edit of the manuscripts; YX, data analysis, review and edit of the manuscripts; JL, data analysis, review and edit of the manuscripts; XZ, data analysis, review and edit of the manuscripts; QC, confirming colposcopy data, data analysis, review and edit of the manuscripts; LS, confirming colposcopy data, data analysis, review and edit of the manuscripts; XT, conceptualization, data analysis, review and editing of the manuscripts; CZ, Project supervision and conceptualization, data analysis, review and editing of the manuscripts. All authors contributed to the article and approved the submitted version.
